# An Affordable Open-Source Turbidimeter

**DOI:** 10.3390/s140407142

**Published:** 2014-04-22

**Authors:** Christopher D. Kelley, Alexander Krolick, Logan Brunner, Alison Burklund, Daniel Kahn, William P. Ball, Monroe Weber-Shirk

**Affiliations:** 1 Department of Geography and Environmental Engineering, Johns Hopkins University, 3400 N. Charles St, Ames 313, Baltimore, MD 21218, USA; E-Mails: lbrunne4@jhu.edu (L.B.); aburklu1@jhu.edu (A.B.); dkahn7@jhu.edu (D.K.); bball@jhu.edu (W.P.B.); 2 Sibley School of Mechanical and Aerospace Engineering, Cornell University, 108 Upson Hall, Ithaca, NY 14853, USA; E-Mail: amk283@cornell.edu; 3 School of Civil and Environmental Engineering, Cornell University, 220 Hollister Hall, Ithaca, NY 14853, USA; E-Mail: monroe.weber-shirk@cornell.edu

**Keywords:** turbidity, affordable water treatment monitoring, open-source hardware, Arduino

## Abstract

Turbidity is an internationally recognized criterion for assessing drinking water quality, because the colloidal particles in turbid water may harbor pathogens, chemically reduce oxidizing disinfectants, and hinder attempts to disinfect water with ultraviolet radiation. A turbidimeter is an electronic/optical instrument that assesses turbidity by measuring the scattering of light passing through a water sample containing such colloidal particles. Commercial turbidimeters cost hundreds or thousands of dollars, putting them beyond the reach of low-resource communities around the world. An affordable open-source turbidimeter based on a single light-to-frequency sensor was designed and constructed, and evaluated against a portable commercial turbidimeter. The final product, which builds on extensive published research, is intended to catalyze further developments in affordable water and sanitation monitoring.

## 1. Introduction

Turbidity refers to the cloudiness of a fluid medium and is quantified by the intensity of light scattered by particles suspended in the medium [1]. For the purposes of water quality monitoring, the American Water Works Association defines turbidity as a “nonspecific measure of the amount of particulate material in water” including “clay, silt, finely divided organic, and inorganic matter” [2]. The colloid-sized particles (with diameters roughly between one nanometer and one micrometer) principally responsible for turbidity in water may have high specific surface area, and often represent the majority of chemical contamination in a water supply as they can adsorb water quality contaminants such as heavy metals or pesticides [3]. Perhaps more importantly, such particles provide microscopic refuges for pathogens, absorb and scatter ultraviolet light (rendering UV light less effective as a disinfectant), and often have a high fraction of natural organic matter, which can consume the oxidizing power of chemical disinfectants such as chlorine and ozone and may form toxic by-products in the process [4]. The particles that cause turbidity can thus significantly impair the effectiveness of disinfection processes for drinking water treatment and turbidity is therefore recognized, both in the relevant engineering literature and in regulations promulgated by the Environmental Protection Agency (EPA), as a principal indicator of the cleanliness and potability of water [5–7].

Turbidity is most commonly quantified by the Nephelometric Turbidity Unit (NTU), or the equivalent Formazin Nephelometric Unit (FNU). Nephelometry refers to the process of aiming a beam of light at a sample of liquid and measuring the intensity of light scattered at 90 degrees to the beam. Further, the NTU/FNU scale is defined in nephelometric analysis by comparison against reference colloidal suspensions of the polymer formazin [8]. The human eye can detect turbidity levels down to roughly 5 or 10 NTU. Small samples of water with turbidity lower than this will appear clear to the human eye, however such samples may still contain a concentration of colloidal particles sufficient to impair disinfection efforts and may carry a load of contaminants or pathogens sufficient to cause serious human illness [7]. Current EPA regulations stipulate that conventionally treated surface water in the USA must be regularly sampled for turbidity, that only 5% of samples in a given month may show turbidity greater than 0.3 NTU, and that no sample may show turbidity in excess of 1.0 NTU [9]. Other countries employ different standards, and the World Health Organization (WHO) recommends that turbidity levels be less than 1.0 NTU prior to disinfection [10]. The gap between human visual detection limits and safe exposure limits has led to the development of electronic devices that employ nephelometry to measure turbidity. Standards for the design and calibration of these devices, which are commonly known as turbidimeters (or nephelometers), have been specified in EPA Method 180.1 [8] and International Standards Organization regulation ISO 7027 [11].

**Figure 1.**
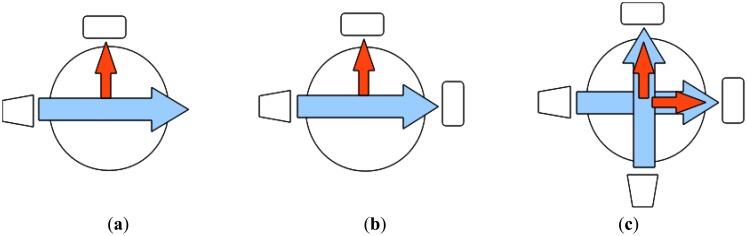
Common turbidimeter design patterns: (**a**) single-beam, (**b**) ratio, (**c**) modulated four-beam. Components: light source (trapezoid), liquid sample (circle), detector (rectangle), transmitted light (large arrow), scattered light (small arrow).

Most turbidimeters contain: (1) a light source that is directed through a liquid sample; (2) a chamber to hold the liquid sample; and (3) one or more photodetectors placed around the chamber. Three archetypal turbidimeter design patterns are diagrammed in Figure 1. A single-beam turbidimeter only measures scattered light, while ratio and modulated four-beam turbidimeters also measure transmitted light (the latter alternating between two light sources). Single-beam turbidimeter designs have upper detection limits that are inherently lower than those of ratio or modulated four-beam turbidimeter designs, since the intensity of scattered light varies non-linearly with turbidity. That is, in very clear water an increase in turbidity will result in more light scattering, but for sufficiently turbid water the addition of more colloidal particles may increase multiple scattering such that a scattered-light photodetector may report an apparent *decrease* in turbidity. Ratio and modulated four-beam turbidimeters normalize readings of scattered light using readings of transmitted light; series of these normalized values can remain linear even at very high turbidities [7].

Commercial turbidimeters employ precision optics and electronics to detect turbidity readings as low as 0.02 NTU in samples of varying color and chemical composition (in accordance with EPA and ISO certification protocols). Handheld commercial models, capable of analyzing a sample manually loaded in a quartz cuvette, typically cost upwards of $600. Automated (inline) turbidimeters, capable of intermittently analyzing samples from a moving column of water and relaying results to a computer or data-logger, typically cost upwards of $2,000. In many areas of the world, communities may not have the fiscal resources to purchase and maintain devices with costs this high, or water treatment monitoring may not be a sufficient priority to justify this expense.

A rich body of peer-reviewed literature has served to document novel turbidimeter designs. Researchers have developed devices for diverse applications such as quality control for the food industry [12,13], field measurements of suspended solids [14,15], and dynamic operation of small appliances [16,17]. Others have explored low-cost sensor designs [18,19] and incorporated wireless connectivity for distributed real-time turbidity monitoring [20]. Many researchers have developed or reviewed devices for low-cost monitoring of turbidity in drinking water [19,21–28]. Our analysis of these devices, in the context of low-resource communities in developing regions, suggests important remaining needs.

Globally, there are billions of people in innumerable communities who are consuming drinking water that is inadequately treated and poorly monitored. Many of these communities have sources of water that are variable in quality and highly susceptible to turbidity spikes (e.g., as the result of sediment run-off in storm events), and many are in areas that lack reliable electricity transmission. There are many promising novel turbidimeter designs published in peer-reviewed literature. We submit that for the context of water monitoring in low-resource and developing communities, a turbidimeter intended for broad adoption and use should meet the following general criteria: (1) the device should run on DC power provided by commercially common batteries (e.g., AA) and provide weeks to months of regular use on one change of batteries; (2) device costs should be detailed and should be kept to an absolute minimum; (3) device should demonstrate a high degree of measurement precision, and sufficient accuracy to detect small changes in turbidity—especially over the critical range of 0–10 NTU where turbidity is invisible to the human eye; (4) device performance should be thoroughly tested across its detection range; (5) the device should be able to measure down to well below 1.0 NTU to address WHO turbidity guidelines, and up to well over 100 NTU (surface water may reach 1,000 NTU or more during a storm event); (6) device construction and programming should be well detailed so that non-experts can independently construct their own version, and tailor or improve existing designs. We further submit that a turbidimeter has not yet been detailed that meets these criteria for this important use case. Informed by prior published turbidimeter designs, we undertook the design, fabrication, and evaluation of a low-cost turbidimeter that meets all of the above criteria and can be employed for water treatment and distribution monitoring in low-resource communities. As further elaborated in following sections, we believe that our developed instrument offers an important improvement over prior models, with potential for immediate application at the pilot scale.

## 2. Experimental Section

### 2.1. Design

**Figure 2.**
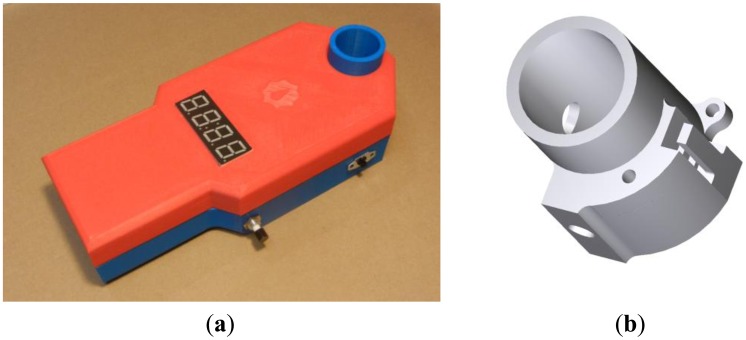
Open-Source Turbidimeter: (**a**) external view, (**b**) image of cuvette holder.

An open-source turbidimeter (see Figure 2a) was built using off-the-shelf electronic components and 3D-printed hardware. The circuit design employs an 8-bit, 20 MHz microprocessor (Model ATMega328P-PU; Atmel, San Jose, CA, USA). The microprocessor was programmed in the C-based Arduino language. The principal housing components—a two-part case and a cylindrical cuvette holder—were made with a commercial 3D-printer (Model Replicator 2×; MakerBot, Brooklyn, NY, USA), although an open-source printer could have also served the purpose. The build envelope of the case measures 205 mm long, 91 mm wide, and 55 mm tall. The cuvette holder houses a near-infrared (860 nm) light emitting diode (LED) and a light-to-frequency sensor (Model TSL230R; TAOS, Plano, TX, USA), placed 90 degrees apart in a “single-beam” design (Figure 1a). The light-to-frequency sensor outputs an electrical pulse train with frequency corresponding to the intensity of detected light [29]. The microprocessor sums pulse counts from the sensor in one-second intervals, and converts these sums to turbidity values using an empirically determined calibration routine (detailed below) stored in persistent memory. Light-to-frequency sensors have been noted as potentially suitable photodetectors in two patents for novel turbidimeter designs [16,17]. The TSL230R in particular has been used to provide turbidity sensing for process control in dishwashers [30], and to determine the biological oxygen demand (BOD) of aqueous solutions [31]. To our knowledge this study represents the first publicly available peer-reviewed characterization of an affordable turbidimeter based on a light-to-frequency sensor.

**Figure 3.**
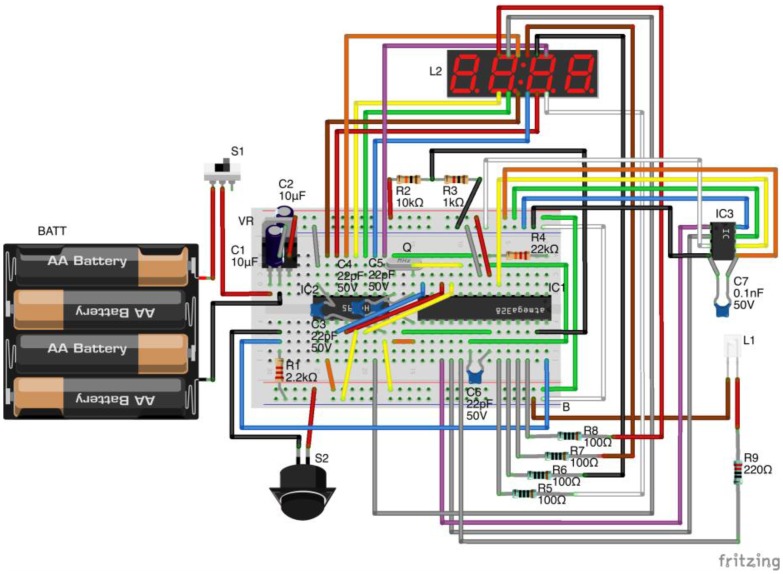
Wiring diagram for the open-source turbidimeter.

Data are displayed on an inexpensive four-digit, seven-segment display panel. The device is powered by four AA batteries and has a sliding power switch and a momentary contact push button on its exterior to initiate sampling and device re-calibration. Battery drain tests indicate that the device can handle hourly sampling for three months on four alkaline AA batteries. This open-source turbidimeter can be built using parts valued at less than $25 and with approximately 3 h of labor. The model we used for these experiments, which employs various hardware conveniences for ease of experimentation (such as a solderless breadboard) has parts costing roughly $35 and can be constructed in 45 min. All schematics and code required to build this open-source turbidimeter are provided in the Supplementary Materials section; the code is copyrighted for public use through the GNU GPLv3 license. The electronic components of the open-source turbidimeter are depicted in Figure 3 and described in detail in the Supplementary Materials.

### 2.2. Calibration and Validation

To convert sensor output to report turbidity, it was necessary to empirically match the sensor's pulse train frequency to corresponding NTU values, and to store this calibration routine in persistent memory of the microprocessor. EPA Standard Method 180.1 states that turbidimeters should be calibrated against aqueous suspensions of the polymer formazin, or an approved formazin alternative [8]. Because formazin is a hazardous chemical that is relatively difficult to use on a routine basis, an alternative was sought. To avoid the extensive cost of purchasing commercially available formazin alternatives (which must be purchased at specified turbidities because they become unstable if diluted), we created a series of 25 stable colloidal suspensions by diluting hydrophilic cutting oil with distilled water, following the approach previously employed and reported by [21].

For calibration of the open-source instrument, each of the 25 cutting oil suspensions was stored in a quartz cuvette and measured eight times with the open-source turbidimeter and eight times with a commercial ratio-based turbidimeter purchased as the standard of comparison for this experiment (MicroTPI model; HF Scientific, Fort Meyers, FL, USA). Concentrations, measured with the commercial instrument, ranged from roughly 0.01 to 1100 NTU. Averaged readings from the open-source turbidimeter for each cutting oil suspension were regressed on averaged readings from the commercial device to develop the calibration curve. Since a primary objective of this experiment is to affordably replicate the behavior of a commercial turbidimeter, individual readings of cutting oil suspensions taken with the open-source turbidimeter were transformed with the calibration curve, and compared to averaged readings from the commercial turbidimeter—these averaged readings from the commercial turbidimeter taken as surrogates for the true turbidity values of the 25 suspensions. The slope and intercept constants of this calibration routine were programmed into the microprocessor of the open-source instrument, and the commercial and calibrated open-source turbidimeters were then tested against five reference turbidity standards (0.02, 1, 10, 100, 1000 NTU, respectively) of an EPA-approved formazin alternative (StablCal, purchased from Fisher Scientific, Pittsburgh, PA, USA). Each reference turbidity standard was measured eight times with each of the turbidimeters. All suspensions and standards were re-measured after 24 h to test for colloidal stability.

## 3. Results and Discussion

### 3.1. Data Summary

**Figure 4.**
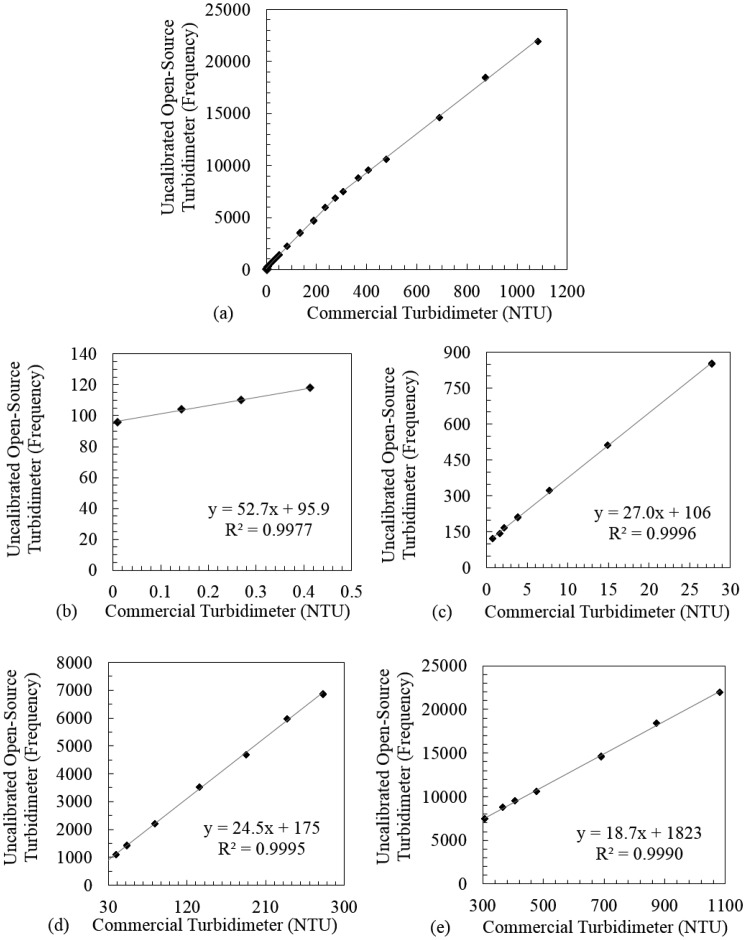
Comparison of averaged open-source turbidimeter and commercial turbidimeter measurements of 25 cutting oil suspensions, overall (**a**) and in four sub-regions: (**b**) 0–0.5 NTU; (**c**) 0.5–30 NTU; (**d**) 30–300 NTU; (**e**) 300–1,100 NTU.

Calibration data from the two instruments are presented in Figure 4. Two of the 200 commercial turbidimeter readings in the calibration dataset were discarded because they were implausibly high for the given sample. The dataset is monotonic across the range of investigation (see Figure 4a) and is approximated well by four linear regressions connected by three transition points (see Figure 4b–e). These transition points were visually selected, and are discussed further below.

**Figure 5.**
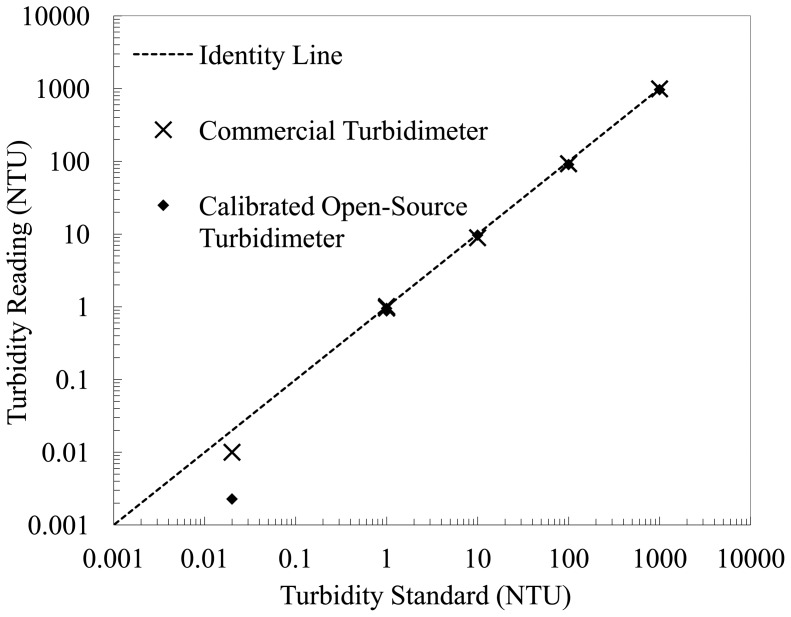
Commercial and open-source turbidimeter measurements of five non-formazin turbidity standards.

One regression line (Figure 4b) covers four concentrations of cutting oil suspension; the other three regression lines (Figure 4c–e) cover seven concentrations each. The regression lines fit the observed values very well above 0.5 NTU (R^2^ ≥ 0.9990), and nearly as well below 0.5 NTU (R^2^ = 0.9977); slope and intercept values are given in Figure 4. Regression residuals were within ± 5% for all data points (and within ± 3% for 23 out of 25 data points). The open-source turbidimeter was thus calibrated, and the calibrated data points were compared to averaged commercial turbidimeter readings to assess data spread. Out of 200 measurements of the open-source device, 192 lie within ± 3% or ± 0.3 NTU (whichever is larger) of the averaged measurement of the commercial device for the respective cutting oil suspension. The remaining eight measurements lie within ± 3.5%, and all measurements for the four suspensions under 0.5 NTU are within ± 0.01 NTU. There were no spatial patterns in the residuals of any regression line, and all *p*-values were less than 0.001. Generally, precision scaled negatively with turbidity value, and accuracy was worse near the transition points between regression lines. The open-source turbidimeter was also tested without a sample in the cuvette holder; both with the light source turned on (frequency: 168 Hz) and turned off (frequency: 0 Hz). After thus calibrating the open-source turbidimeter, both this instrument and the commercial device were used to measure five EPA-approved non-formazin turbidity standards, eight times each, with results shown in Figure 5. The mean, standard deviation, and root-mean-square error of each set of measurements are presented in Table 1.

### 3.2. Discussion

The analysis indicates that the open-source turbidimeter provides a reasonable approximation of results given by a commercial handheld model over the range of 0–1,000 NTU. This is remarkable given that the open-source device can be built for roughly 4% of the cost of the commercial model. Construction requires only a rudimentary knowledge of electronics and access to basic tools and a soldering iron. Since the open-source turbidimeter uses common low-cost electronics components (no part over $6 and only three above $2; see Supplementary Materials), the device can be affordably repaired by owners with access to spare parts. As the construction and improvement of the prototype turbidimeter is an open-source endeavor, complete instructions and parts lists are hosted online at [32] and updated frequently. One important update to the open-source turbidimeter incorporated after these experiments is an internal temperature sensor (LM35; Texas Instruments, Dallas, TX, USA) to measure ambient temperature changes (which can be significant outside of a climate-controlled laboratory) and firmware edits to compensate for thermal effects on the relative intensity of the LED.

Both the open-source and commercial turbidimeters detected the lowest turbidity standard to within ± 0.02 NTU (as stipulated by EPA certification requirements), however it should be noted that random thermal fluctuations can induce apparent turbidity and influence measurements of turbidities this low. A logical next step in this research will be to better characterize performance of the open-source turbidimeter over the range of 0–1 NTU using more EPA-approved non-formazin standards. It is likely though that detection accuracy and precision in this range are of relatively minor concern for communities that are struggling, both financially and technically, to meet stringent turbidity standards. This unfortunate reality is reflected in the relatively high turbidity limits set by many developing countries—e.g., India at 1 NTU [33], and Honduras at 5 NTU [34].

Both the open-source and commercial turbidimeters are imperfect devices. As the open-source device is calibrated against the commercial model, the uncertainty of the commercial model (± 2% or ± 0.1 NTU for 0–500 NTU, ± 3% for 500–1,000 NTU) should affect the accuracy of the open-source model; a source of error we have attempted to minimize by averaging replicate readings of the commercial model. The evaluation of both devices with EPA-approved non-formazin standards of known turbidity is thus an important external validation, but the relative agreement of the two devices is the most important message. Thus while the calibrated open-source turbidimeter appears to outperform the commercial turbidimeter in detecting the value of the 10 NTU standard, this should be taken as coincidental—logically the open-source turbidimeter cannot best the source of its calibration. It is worth noting that both devices measured values lower than the stated values of the turbidity standards in all cases. It is possible that the turbidity standards, although newly purchased from a reputable vendor, may have degraded slightly since formulation. It is also possible that the commercial turbidimeter, although newly purchased from a reputable vendor and calibrated in the factory, may have been slightly off. All cutting oil suspensions and turbidity standards were re-measured after 24 h; none showed drift beyond 0.5% or 1.0 NTU (whichever is smaller) of the respective averaged original readings.

The choice of using multiple regression regions to characterize the calibration dataset presented in Figure 4 was motivated by a slight non-linearity in the dataset (visually most apparent at roughly 300 NTU; see Figure 4a). This may be due to the fact that the open-source turbidimeter uses a single-beam design while the commercial model uses a ratio design, since the linear performance of a single-beam turbidimeter—using only a scattered-light detector—necessarily diminishes with increasing turbidity earlier than that of a ratio turbidimeter, which normalizes scattered light readings with transmitted light readings. The transition values which ensure continuity of the four-part regression equation (0.4 NTU, 26.4 NTU, and 287.8 NTU, respectively) differ slightly from the transition points chosen before regression analysis; the former are used in the calibration function of the software (Supplementary Materials). Exploring the response of the open-source turbidimeter to precisely measured formazin dilutions, and in particular assessing the maximum value of the device's performance range, are important next steps. Still, the evidence presented here indicates that a single-beam turbidimeter employing a light-to-frequency sensor can usefully measure turbidity over a range of 0–1,000 NTU, provided that multiple linear regression ranges are used to convert raw sensor data to turbidity values.

The Supplementary Materials section presents the full set of instructions for assembling the device described in this work, which we have intended to serve as an affordable turbidimeter suitable for basic water-quality monitoring. We believe that this may be the first public and open-source design that is so completely described and evaluated, and we hope that it may thus be made accessible to individuals and communities that are unable to otherwise afford adequate turbidimeters. We do note that, during the course of this work, Anzalone *et al.* published the details of a $50 Arduino-based colorimeter that incorporates the TSL230R light-to-frequency sensor for determination of the biological oxygen demand (BOD) of aqueous solutions, and that these authors also mentioned the possible application of the sensor for affordable nephelometry [31]. We are pleased to confirm their hypothesis, while also achieving significant cost reductions relative to their prototype design (e.g., by replacing commercially purchased, fully assembled circuit boards with comparable minimalist and lower-cost components). The field of low-cost open-source hardware alternatives for science and engineering is rapidly expanding (e.g., in optics [35], chemical synthesis [36], and ultrasonic sensing [37]; see [38] and [39] for broader introductions), and we are glad to extend this range of alternatives to include affordable nephelometric sensing.

### 3.3. Future Work

The data presented above (particularly the y-intercept value of Figure 4(a), and the relatively large frequency measurement of the open-source turbidimeter without a cuvette inserted) suggest that some amount of light is leaking directly from the LED to the sensor. We are currently investigating low-cost insulation strategies to best reduce this leakage. We note that calibration curves of the type presented in Figure 4 will always have non-zero y-axis intercepts, due to random thermal fluctuations. Still, there is likely significant room for noise elimination in the current open-source turbidimeter. It should also be noted that as light-to-frequency sensors such as the TSL230R essentially perform integer truncation on an approximately continuous photon-scattering process, increasing the sampling period may help to differentiate similar samples at sufficiently low turbidities. It will be useful to investigate how the sampling period may be varied (within a user-friendly time limit of 15–30 s) to improve detection at very low turbidities. Future work will also include evaluating the possibility of affordably implementing a ratio or modulated four-beam design to improve detection accuracy and range.

We are currently designing an inline version of the open-source turbidimeter, which will feature an immersible probe rather than a cuvette holder integrated into the main device housing, to better facilitate continuous collection of water quality data. Mindful that proper management and monitoring of water resources requires not only timely collection of data but also the ability to communicate those data with stakeholders, we have designed versions of the open-source turbidimeter with an integrated GSM modem (hardware and software described in Supplementary Materials). This addition allows the turbidimeter to communicate with a web server via wifi (GPRS) and cellular network (GSM). For areas with basic cell phone access but lacking wifi (a common situation in rural areas), we have implemented an SMS syntax that allows the open-source turbidimeter to communicate with a web server by sending text messages to an SMS gateway (such as Twilio, or the open-source tool FrontlineSMS). The prototype GSM-enabled open-source turbidimeter can format and interpret a wide array of commands in properly formatted SMS messages, and supports basic password authentication. This functionality has been used to facilitate two-way communication between the turbidimeter and a server, allowing the turbidimeter operator to automatically communicate water quality data to the web (example given in Supplementary Materials) and to receive intermittent calibration reminders. We have also tested the use of a GSM-enabled turbidimeter to function as a basic messaging switchboard. The operator of the turbidimeter can securely store basic contact information in the turbidimeter's persistent memory via SMS command, and people viewing uploaded water quality data on the web can communicate with the operator (via the server-to-modem communication backbone) without either side divulging personal contact details to the other. Adding GSM/GPRS functionality to the open-source turbidimeter raises the hardware costs by $40 to $100 depending on the modem selected, but the basic SMS syntax we have designed can be used to communicate water quality data to the web from any cellphone with no additional hardware required. Currently, several non-electric water treatment plants in Honduras are utilizing this SMS syntax to communicate water quality data twice daily to the web (data available at [40]).

## 4. Conclusions/Outlook

In this paper we have characterized a novel turbidimeter suitable for basic water quality monitoring. Our analysis indicates that the turbidimeter detects turbidity with range, accuracy, and precision fairly comparable to those of a much more expensive, portable commercial device. This turbidimeter has been designed to be reliable, portable, sensitive over the typical turbidity range of natural water bodies, and highly affordable. We have developed communication protocols—and specified optional hardware and software—to help ensure that collected data can be shared in a timely manner with stakeholders. To encourage adaptation and improvement of this device we have designed it to be simple to repair, provided full instructions for assembly, and licensed it as an open-source technology.

Next steps in this line of research include improving detection of very low levels of turbidity, ruggedizing the device, and developing a reliable inline version. There is an urgent need for effective real-time water quality monitoring in low-resource communities around the world, and flexible, low-cost turbidity monitoring is a key part of meeting this need. It is our ardent intention that the design and characterization of the turbidimeter described here will help speed the proliferation of low-cost water quality monitoring technology and will better enable engineers, supervisors, and water technicians in all communities to make timely, informed assessments of water quality.

## Supplementary Material



## Figures and Tables

**Table 1.** Mean, standard deviation (SD), and root-mean-square error (RMSE) of commercial and open-source turbidimeter readings of five non-formazin turbidity standards.

**Turbidity Standard (NTU)**	**Measure**	**Commercial Turbidimeter (NTU)**	**Interpolated Open-Source Turbidimeter (NTU)**
1,000	MEAN	992	968
	SD	0.68	1.05
	RMSE	7.60	31.5

100	MEAN	92.6	90.4
	SD	0.22	0.07
	RMSE	7.41	9.54

10	MEAN	8.90	9.68
	SD	0.01	0.00
	RMSE	1.10	0.33

1	MEAN	0.98	0.93
	SD	0.02	0.03
	RMSE	0.03	0.08

0.02	MEAN	0.01	0.00
	SD	0.00	0.00
	RMSE	0.01	0.02
