# Supplementary material for: An Affordable Open-Source Turbidimeter
Source: Sensors (Basel). 2014 Apr 22;14(4):7142–55. doi: 10.3390/s140407142 (PMC4029670; doi:10.3390/s140407142)
Supplement: Supplementary file 1 [file sensors-14-07142-s001.pdf]

OPEN ACCESS

sensors

ISSN 1424-8220

www.mdpi.com/journal/sensors

Supplementary Material

An Affordable Open-Source Turbidimeter. *Sensors* 2014, *14*, 7142-7155

Christopher D. Kelley [1,]*, Alexander Krolick [2], Logan Brunner [1], Alison Burklund [1], Daniel Kahn [1], William P. Ball [1] and Monroe Weber-Shirk [3]

[1] Department of Geography and Environmental Engineering, Johns Hopkins University, 3400 N. Charles St, Ames 313, Baltimore, MD 21218, USA; E-Mails: lbrunne4@jhu.edu (L.B.); aburklu1@jhu.edu (A.B.); dkahn7@jhu.edu (D.K.); bball@jhu.edu (W.P.B.)

[2] Sibley School of Mechanical and Aerospace Engineering, Cornell University, 108 Upson Hall, Ithaca, NY 14853, USA; E-Mail: amk283@cornell.edu

[3] School of Civil and Environmental Engineering, Cornell University, 220 Hollister Hall, Ithaca, NY 14853, USA; E-Mail: monroe.weber-shirk@cornell.edu

* Author to whom correspondence should be addressed; E-Mail: ckelle13@jhu.edu; Tel.: +1-410-516-7092; Fax: +1-410-516-8996.

S1. Introduction

This Supplementary Material provides additional detail on the construction of the device outlined in the article “An Affordable Open-Source Turbidimeter”. The sections below detail the necessary parts and tools, wiring, assembly, and microcontroller programming. Readers are encouraged to contact the corresponding author with any questions about the construction, operation, or expansion of this device.

S2. Component List

All components of the open-source turbidimeter are indicated below, along with potential distributors. Indicated costs are for individual pieces and do not include shipping and handling or taxes. Please note that the parts list and diagrams presented below represent the design for the open-source handheld turbidimeter evaluated in the main article, and differ slightly from the most current model hosted at https://github.com/wash4all/open-turbidimeter-project. Please visit this site for updated parts lists and assembly instructions.

Table S1. Components of the open-source turbidimeter.

**Basic Unit Parts**					
Qty	Name	Description	Vendor	Part #	Unit Price
1	IC1	8-bit Microcontroller	Mouser Electronics	ATMEGA328P-PU	2.24
1	IC2	595 Shift Register	Mouser Electronics	SN74HC595N	0.63
1	IC3	TSL230R Sensor	C-Stamp	CS454000	5.94
1	Q	16MHz 18pF HC49S Crystals	Mouser Electronics	HC49US-16.000MABJ-UB	0.58
1	VR	5V Voltage Regulator	Mouser Electronics	LM7805CT	0.69
2	C1,2	10uF Capacitor	Mouser Electronics	UKL1C100MDD1TA	0.15
4	C3-6	22pF Capacitor	Mouser Electronics	1C10C0G220J050B	0.015
1	C7	0.1nF Capacitor	Mouser Electronics	140-50S5-101J-RC	0.06
1	R1	1/4 Watt 2.2 kOhm Resistor	Mouser Electronics	MF1/4DCT52R2201F	0.06
1	R2	1/4 Watt 10 kOhm Resistor	Mouser Electronics	MF1/4DCT52A1002F	0.06
1	R3	1/4 Watt 1 kOhm Resistor	Mouser Electronics	MFS1/4DCT52R1001F	0.13
1	R4	1/4 Watt 22K Ohm Resistor	Mouser Electronics	MF1/4LCT52R223J	0.07
4	R5-8	1/4 Watt 100 Ohm Resistor	Mouser Electronics	MF1/4DC1000F	0.06
1	R9	1/4 Watt 220 Ohm Resistor	Mouser Electronics	MF1/4CCT52R2000F	0.14
1	B	Vectorboard *	Electrodragon	5205883	0.38
1	S1	Power Switch	Electrodragon	4201001	0.80
1	S2	Black Button	Mouser Electronics	104-0013-EVX	0.91
1	L1	860nm Infrared LED	Mouser Electronics	W53SF6C	1.12
1	L2	7-Segment Display	Electrodragon	4103417	2.00
1	BATT	4xAA Battery Holder	Mouser Electronics	12BH348-GR	1.15
1	-	M/F Jumper Wires (22 count)	Electrodragon	5101606	1.20
1	-	Printed Cuvette Holder**	Octave	3DP-JC-ABK1.7-10	0.92
1	-	Printed Case Exterior**	Octave	3DP-JC-ABK1.7-10	4.88
Total Basic Unit Cost					$24.56
**Cuvettes**					
2		Glass Cuvette–27mm diameter ***	(Various)	--	~0.50
OR		Quartz Cuvette (3 pack) ***	Fisher Scientific	14-376-167	47.60
**Other Communication Parts**					
1		GSM Modem	Mouser Electronics	A000043	88.95

* For ease of use, a 30-row breadboard with side power channels (Electrodragon, #5205103, $2.50) was used in place of a vectorboard for tests detailed in "An Affordable Open-Source Turbidimeter"; **These parts were printed using Black ABS 1.75mm filament purchased from the distributor ($31.00/kg).*** See note in step 4 of section S6, about glass cuvettes *versus* quartz cuvettes.

S3. Structural Components

The open-source turbidimeter case consists of four different parts: (1) bottom, which includes ports for button and on/off switch; (2) cuvette holder, which houses the sensor and light source and holds the glass cuvette during device operation; (3) top, which has openings for the seven-segment LED display and the top of the cuvette holder; and (4) battery lid, which slides out from the case assembled case top and bottom to expose the battery holder. Electronic files of these objects (in STL format) are available upon request. We wish to note that any container that provides good shielding from incidental light may suffice (e.g., a nested pair of cardboard boxes).

Figure S1. Image of the open-source turbidimeter with major dimensions given. The four smaller images (left to right) are the case lid (top left), the case base (bottom left), the battery lid (top right), and the cuvette holder (bottom right). All dimensions are in millimeters.

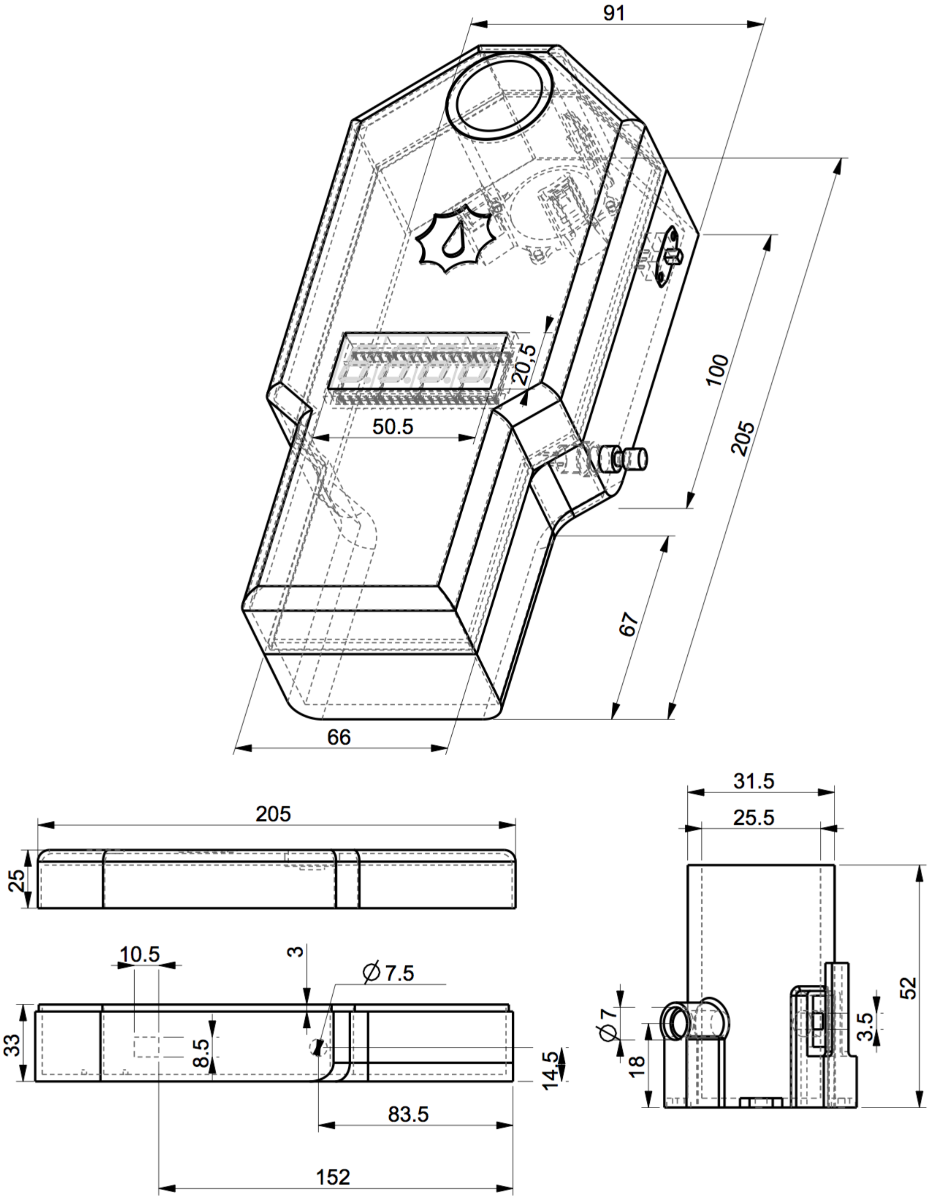

S4. Tools

A utility knife, soldering iron, and solder are required for assembly of the open-source turbidimeter, and safety goggles are recommended. To replicate the printed four-part case, one must have access to a 3D-printer with black ABS filament. The microprocessor can be programmed via a USB-to-Serial adapter (Mouser carries one [#A000059] for $14.95) or with an Arduino (as described at http://arduino.cc/en/Tutorial/ArduinoToBreadboard). Electrical tape is very helpful (for grouping wires together), as are a pair of wire strippers.

S5. Turbidimeter Breadboard and Schematic

The microprocessor and connected components of the open-source turbidimeter are depicted below in Fritzing (Figure S2) and EAGLE formats (Figure S3). Figure S2 depicts what the device looks like internally, while Figure S3 is a more formal schematic.

Figure S2. Depiction of the internal components of the open-source turbidimeter (components labels cross-referenced in Table S1 and Figure S3).

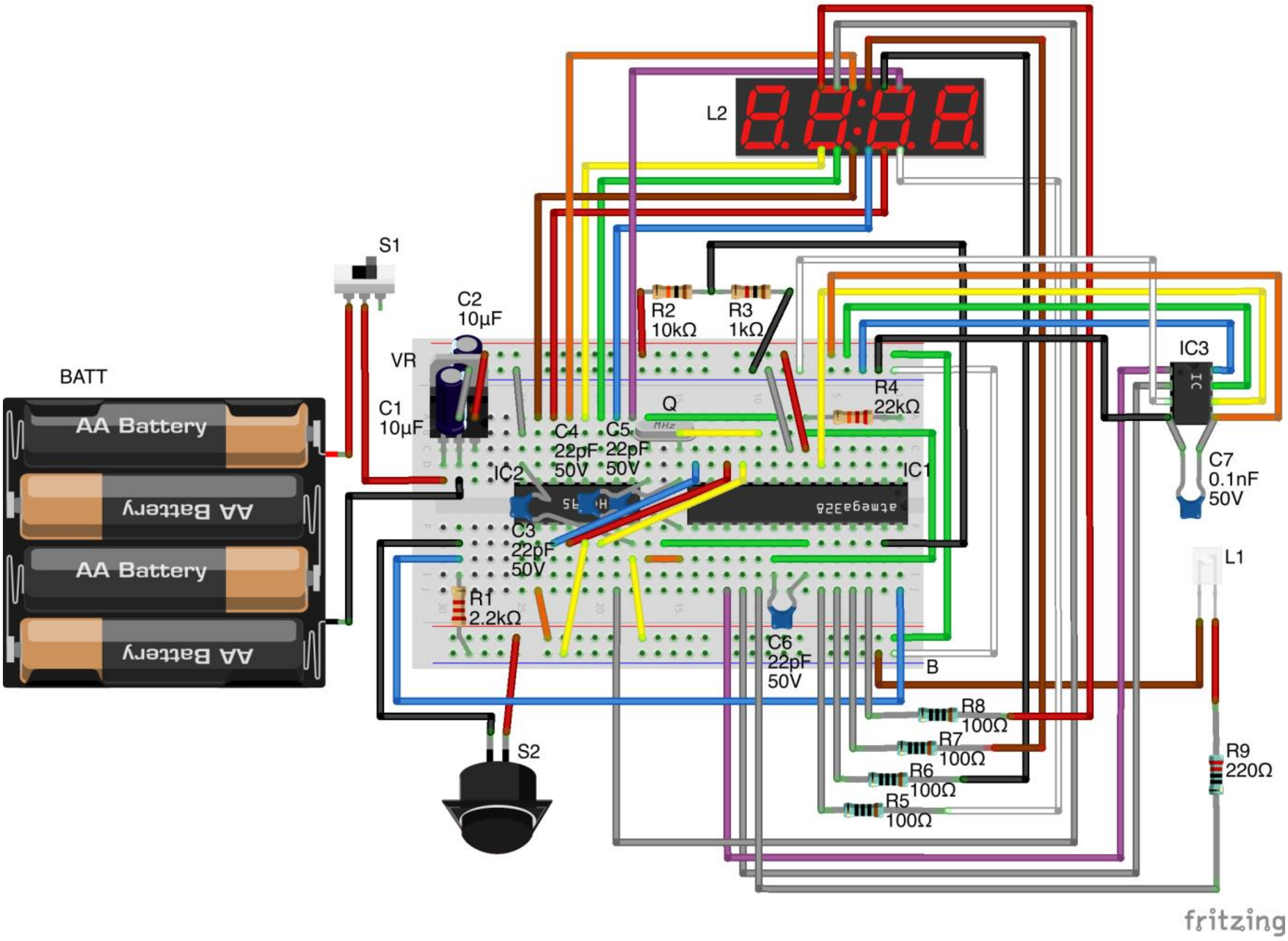

Figure S3. Schematic of the circuit board for the open-source turbidimeter (components labels cross-referenced in Table S1 and Figure S2).

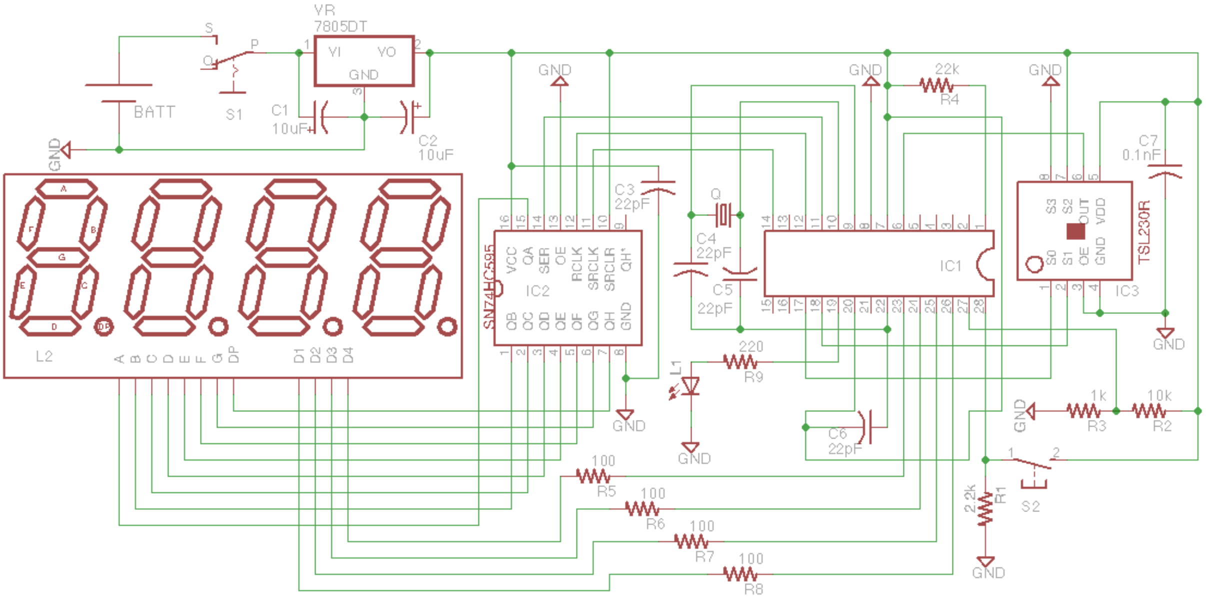

S6. Basic Assembly Instructions

1. Gather the components listed in Section S1, and the tools listed in Section S3.
2. Print the parts described in Section S2 (or devise your own light-shielding case).
3. Wire together the internal components of the open-source turbidimeter according to Figures S2 and S3. Here are some key points:
   a. It's hard to see some connections of the circuit, such as the wiring of the voltage regulator (VR1), on Figure S2. The Arduino website has a well-illustrated tutorial on wiring the ATMega328P microprocessor (http://arduino.cc/en/Main/Standalone) that the reader may find useful.
   b. The semi-circles on the microprocessor (IC1) and shift register (IC2) indicate orientation of these components. It is vital that chips are aligned properly using these guides. Wiring them in the opposite orientation will destroy the chips once voltage is applied! Please note that the semi-circle on the TSL230R sensor in Figure S2 is merely a visual aid, and is not present on the actual chip.
   c. The pins of the seven-segment display are diagrammed by the distributor Electrodragon (http://www.electrodragon.com/w/index.php?title=7-Segment_Display). The linked document maps pin placement to function and representation in Figure S3 (e.g., the bottom left pin on the seven-segment display is pin 12, which maps to segment E of the display, which connects to port QE on the shift register).
   d. Resistors R2 and R3 combine to form a voltage divider, which is used to measure the voltage provided by the batteries. To achieve useful results, use resistors with tolerance of 1% or less. Tolerance levels of 5% are suitable for the other resistors in the device.
   e. The internal components list generally should be followed closely, however the power switch (S1) and button (S2) can easily be changed to suit the builder's aesthetic.

f. As always, exercise caution. Do not attempt construction of any electrical device without knowledge of proper technique and safety.

4. Test open-source turbidimeter by placing water sample in glass cuvette, inserting glass cuvette into the device, and analyzing.
   Note: The experiments described in the article were conducted with quartz cuvettes, which may improve optical clarity but at great expense (~$16 each). If using borosilicate cuvettes, it is advisable to calibrate the turbidimeter manually, rather than using the calibration constants provided in section S7. When using borosilicate glass cuvettes, it is particularly important to visually inspect the cuvette for scratches, and to always measure turbidity with the cuvette inserted into the sample chamber at the same orientation (this is good advice even with quartz cuvettes). The easiest way to do this is to nick the lid of the cuvette with a knife, and align this nick with the centerline of the turbidimeter case.

S7. Programming

The following program, written in Arduino-C, runs the assembled open-source turbidimeter. Connect the device to a computer using a properly wired USB-to-Serial adapter (this requires the use of an additional 100nf capacitor, see http://arduino.cc/en/Main/USBSerial) or an Arduino board (tutorial given at http://arduino.cc/en/Tutorial/ArduinoToBreadboard). Then install the Arduino programming environment (http://arduino.cc/en/main/software). Open a new "sketch" (empty program) and past the code below into it, staying mindful of any formatting quirks that may result from the article formatting process. Compile the new sketch and upload it to the microprocessor.

The Arduino firmware program ("sketch") relies on separately-distributed libraries for several features. These are the EEPROM and EEPROMAnything libraries for persistent memory, the PinChangeInt library for setting interrupts on the light sensor, and the GSM library for wireless communication. The EEPROMAnything and PinChangeInt libraries must be downloaded separately and imported using the "Sketch > Import Library" menu in the Arduino IDE. The GSM library is already included in the Arduino installation directory (*i.e.*, Program Files on Windows), but requires slight modification to resolve a known conflict with the interrupts-setting functionality of PinChangeInt. After creating a backup copy of the GSM library (found in Libraries folder of Arduino) edit the file named GSM3SoftSerial.cpp. Comment out the code blocks for PCINT0 and PCINT1 that look like:

```
#if defined(PCINT0_vect)
ISR(PCINT0_vect)
{
  GSM3SoftSerial::handle_interrupt();
}
#endif
```

Leave the PCINT2 and PCINT3 sections alone; these are needed for the GSM library to work.

```
/*-------------------------OPEN TURBIDIMETER PROJECT------------------------*/
/*------------------------------ ARDUINO CODE -----------------------------*/
```

```
/*
For a copy of the most up-to-date code, please visit:
https://github.com/wash4all/open-turbidimeter-project

Copyright 2012-2014 Alex Krolick and Chris Kelley

     This program is free software: you can redistribute it and/or modify
    it under the terms of the GNU General Public License as published by
    the Free Software Foundation, either version 3 of the License, or
    (at your option) any later version.

    This program is distributed in the hope that it will be useful,
    but WITHOUT ANY WARRANTY; without even the implied warranty of
    MERCHANTABILITY or FITNESS FOR A PARTICULAR PURPOSE.  See the
    GNU General Public License for more details.

    You should have received a copy of the GNU General Public License
    along with this program.  If not, see <http://www.gnu.org/licenses/>.
*/


// Flags
  boolean debug = false;  // IMPORTANT to update EEPROM, change this to true
  boolean using_modem = false; // if not using a GSM modem, change to false

// Libraries
// NOTE: Be sure to use the libraries included with this sketch
// (by placing them in your Arduino or Sketchbook folder)
  #define NO_PORTD_PINCHANGES
  #define NO_PORTC_PINCHANGES
  #include <GSM.h>
  #include <PinChangeInt.h>
  #include <EEPROM.h>
  #include <EEPROMAnything.h>

// Definitions
  #define PINNUMBER "1111" // PIN Number for SIM card
  #define VERSION_BYLINE "Open Turb Prj\nBIOS v1.9\n2014-02-11\n"
  #define IR_LED   13    // light source
  #define TSL_S1   12    // S1 and S0 are pins on the TSL230R chip
  #define TSL_S0   11
  #define TSL_FREQ  4    // frequency signal from the sensor
  #define BPIN     A5    // external button
    // Gauge voltage by comparison to ATMega328P's internal 1.1v
    // R1 and R2 form a voltage divider, with V_out = V_in * R2 / (R1 + R2)
    #define VPIN     A4    // read voltage from this pin
    #define DIV_R1 10000   // resistance for R1
    #define DIV_R2  1000   // resistance for R2
  #define SAMPLING_WINDOW 1000    // milleseconds between frequency calculations
  #define HIGH_SENSITIVITY  100   // sensitivity settings, set via S0 and S1 pins
  #define MED_SENSITIVITY   10    // ...
  #define LOW_SENSITIVITY   1     // ...
  #define READ_REPS 6    // number of readings the turbidimeter will take per
                         // button press (average is used for reporting)

// Set up GSM library
  GSM gsmAccess;
  GSM_SMS sms;
  boolean notConnected = true;
  char* remoteNum = "14105555555";
  char* selfNum = "15125555555";
  String sNum = "151255555555";

// Global Vars
  int  scale_divider = 2, sensitivity = HIGH_SENSITIVITY;
    // scale_divider must match hardwired TSL_S2 and TSL_S3 settings,
    // sensitivity sets TSL_S0 and TSL_S1 settings
    // see TSL230R user's guide for more information
  int bpress = 1023;
```

```
    //variable for digital mapping of analog button press event
  float div_fact = 1;
    //division factor, to normalize sensor output by voltage level
  long freq_jump_hi = 50000, freq_jump_lo = 4000;
    // (These settings could be used to dynamically set sensor sensitivity,
    // e.g. for detecting very bright light levels)
  unsigned long timer, frequency;
  volatile unsigned long pulse_count = 0;
    //interrupt variable; stores count data coming from sensor
  boolean bpressed = false;
  boolean sufficient_battery = true;
  const int num_displays = 4;
  const int shift_latch = 5;  // RCLK
  const int shift_clock = 8;  // SRCLCK
  const int shift_data = 6;   // SER
    // The above three pins connect the 74HC595 shift register
  const int dispPorts[num_displays] = {A3,A2,A1,A0};
  String language = "english"; //"espanol";

  // Characters: 0-9, '.', '-', blank
  const byte SevenSegNumbers[13] = {B11000000,  B11111001,  B10100100,  B10110000,
                                    B10011001,  B10010010,  B10000010,  B11111000,
                                    B10000000,  B10010000,  B01111111,  B10111111,
                                    B11111111};

  // Characters: rEdYLStO
const byte SevenSegLetters[8] =  {B10101111,  B10000110,  B10100001,  B10010001,
                                    B11000111,  B10010010,  B10000111,  B11000000};

// Use this structure for storing data & retrieving data persistently in EEPROM
  struct config_t{
    int foo;           //example
    long machine_id;   //example
    unsigned long last_calibration_timestamp; // in seconds since 1/1/1970 12:00a
      // define calibration constants for 4 calibration curves
      // y is the lower bound, m is the slope, b is the the y-intercept (y=mx+b)
      // PLEASE NOTE: The calibration curve included in this code has five linear regions instead of four, and is the
      //       result of additional device testing to that reported in the article An Affordable Open-Source Turbidimeter
    float y0, y1, y2, y3, y4,
          m0, m1, m2, m3, m4,
          b0, b1, b2, b3, b4;
  }
  config;

/*---------------------------------------------------------------------------*/
/*--------------------------------FUNCTIONS--------------------------*/
/*---------------------------------------------------------------------------*/
void setup() {

  // Pin I/O settings
    pinMode(TSL_FREQ, INPUT); // light sensor
    pinMode(TSL_S0, OUTPUT);  // light sensor
    pinMode(TSL_S1, OUTPUT);  // light sensor
    pinMode(IR_LED, OUTPUT);  // light source
    pinMode(BPIN, INPUT); // button
    pinMode(VPIN, INPUT); // voltage
    pinMode(shift_latch, OUTPUT); // shift register
    pinMode(shift_clock, OUTPUT); // shift register
    pinMode(shift_data,  OUTPUT); // shift register
    for(int i = 0; i < num_displays; i++){
      pinMode(dispPorts[i], OUTPUT);
      // set display pins to output
    }

  // Startup procedure
    digitalWrite(IR_LED, LOW);  // light source off
    delay(200);
```

```
    turnOffDisplay();
    setSensitivity(sensitivity); // set sensor sensitivity
    timer = millis();
    displayForInterval(-1, "dashes", 1000);

  // Prepare sensors
    if(divisionFactor_TSL230R() < 0){
      sufficient_battery = false;
      displayForInterval(-1, "error", 2000);
    }
    else{
      displayForInterval(-1, "ready", 2000);
      turnOffDisplay();
    }

  if(debug){
    config.foo = 255;
       //EEPROMAnything seems to need the struct to start with a integer in [0,255]
    config.machine_id = 11111111; //example
    config.last_calibration_timestamp = 1390936721;
    config.y0 = 0;
    config.m0 = 0.02876;
    config.b0 = -2.224;
    config.y1 = 87.63;
    config.m1 = 0.039;
    config.b1 = -3.124;
    config.y2 = 276.94;
    config.m2 = 0.04688;
    config.b2 = -5.298;
    config.y3 = 2472.3;
    config.m3 = 0.05223;
    config.b3 = -18.525;
    config.y4 = 6049;
    config.m4 = 0.0721;
    config.b4 = -138.9;
    EEPROM_writeAnything(0, config);
     // Write example calibration settings to EEPROM memory
  }
  else{
    EEPROM_readAnything(0, config);
      // Read calibration data from EEPROM memory
  }
}

void loop() {
  if(sufficient_battery){
    bpress = analogRead(BPIN);
      // check for button press event (0 = pressed)
    if (bpress == 0){
      bpressed = true;
      divisionFactor_TSL230R();
        // read, but discard first reading
      div_fact = divisionFactor_TSL230R();
        // take another reading of voltage divider
      if(div_fact < 0){
        sufficient_battery = false;
      }
      else{
        // Given there is enough battery power for the  sensor,
        // - read sensor value for given number of times,
        // - display the resulting value for 4000 milliseconds,
        // - clear out register pins, for a clean display next
        //   time device is powered off/on.
        // - build a text message and transmit,
        //   if using_modem flag is set to true.
        float reading = takeReadings(READ_REPS);
        displayForInterval(reading, "data",4000);
```

```
        displayForInterval(-1, "clear", 100);

        if(using_modem){
          int msg_len = 140;
          char txtMsg[msg_len];
          String bn, message_text;
          bn = baseNmap(reading);
          message_text = "#cod debug #con xxxxxxxxxxx #mtac " + bn;
          // This command as currently coded will send
          // a coded text message of every reading!!
          openConnection();
          delay(30000);
          sendMessage(selfNum, message_text);
          closeConnection();
        }
      }
    }
    if(bpressed){
      bpressed = false; // reset
      turnOffDisplay();
    }
  }else{
    divisionFactor_TSL230R();
    displayForInterval(-1, "error", 500);
    turnOffDisplay();
    delay(500);
  }
}

/*--------------------------Voltage Meter Functions--------------------------*/
float divisionFactor_TSL230R(){
  float m = .0052;   //slope of sensor's linear response curve
  float vmin = 3.0;  //min operating v of sensor
  float vmax = 5.5;  //max operating v of sensor
  float v100 = 4.9;  //voltage | normalized response of TSL230r = 1.0
  analogReference(INTERNAL);
  delay(200);
  float v = getVoltageLevel();
  analogReference(DEFAULT);
  delay(200);
  if(v < vmin || v > vmax){return -1;}
  else{return 1 - (4.9 - v) * m;}
}

float getVoltageLevel(){
  float sensorValue = analogRead(VPIN); //drop the first reading
  delay(100);
  sensorValue = float(analogRead(VPIN));
  float divider_value = float(DIV_R2) / float(DIV_R1+DIV_R2);
  float voltage = sensorValue/ 1023.0 * 1.1 / divider_value;
    // normalize by max mapping value, internal reference voltage,
    // and voltage divider, respectively.
  return voltage;
}

/*------------------------------SevSeg Functions-----------------------------*/
void turnOffDisplay(){
  for(int i = 0; i < num_displays; i++){
    digitalWrite(dispPorts[i], LOW); //turn off digit pins
    }
}

void DisplayADigit(int dispnum, byte digit2disp){
  digitalWrite(shift_latch, LOW);                    //turn shift register off
  turnOffDisplay();
  shiftOut(shift_data, shift_clock, MSBFIRST, digit2disp);     // perform shift
  digitalWrite(shift_latch, HIGH);                    // turn register back on
```

```
  digitalWrite(dispnum, HIGH);
  delay(2);                                    // for persistance of vision
}

void SevenSegDisplay(float f, String msg){
  if(msg == "data"){
    long powers[6] = {10000, 1000, 100, 10, 1};
    if(f > 9999){f = 9999.0;}  //bounds checks for display
    if(f < 0){f = 0.0;}
    int numeric_scale = 1, pt = -1, start = 0;
      // determine where to put decimal point and leading blank digit, if needed
    if(f > 1000){;}
    else if(f > 100){
      numeric_scale = 10;
      pt = 2;
    }else if(f > 10){
      numeric_scale = 100;
      pt = 1;
    }else if(f > 1){
      numeric_scale = 100;
      pt = 1;
      start = 1;
      DisplayADigit(dispPorts[0],SevenSegNumbers[0]);
    }else{
      numeric_scale = 100;
      start = 2;
      DisplayADigit(dispPorts[0],SevenSegNumbers[12]);
      DisplayADigit(dispPorts[1],byte(SevenSegNumbers[0] | SevenSegNumbers[10]));
    }
    long f2l = long(f * numeric_scale);
    for(int i = start; i < 4; i++){
      if(i == pt){
        DisplayADigit(dispPorts[i],SevenSegNumbers[(f2l% powers[i]) / powers[i+1]] | SevenSegNumbers[10]);
          // perform modulo and integer division calculations to separate digits
          // bit mask with decimal point if needed
      }
      else{
        DisplayADigit(dispPorts[i],SevenSegNumbers[(f2l% powers[i]) / powers[i+1]]);
          //do the above line this way if decimal point not needed for given digit
      }
    }
  }else if(msg == "dashes"){
    for(int i = 0; i < num_displays; i++){
      DisplayADigit(dispPorts[i],SevenSegNumbers[11]);
    }
  }else if(msg == "cycle_dashes"){
    for(int i = 0; i < num_displays; i++){
      DisplayADigit(dispPorts[i],SevenSegNumbers[11]);
      delay(100);
    }
  }else if(msg == "ready"){
    // display best available approximation of "ready" message,
    // in Spanish or English, on seven-segment display
    if(language == "english"){
      DisplayADigit(dispPorts[0],SevenSegLetters[0]);  //r
      DisplayADigit(dispPorts[1],SevenSegLetters[1]);  //E
      DisplayADigit(dispPorts[2],SevenSegLetters[2]);  //d
      DisplayADigit(dispPorts[3],SevenSegLetters[3]);  //Y
    }else if(language == "espanol"){
      DisplayADigit(dispPorts[0],SevenSegLetters[4]);  //L
      DisplayADigit(dispPorts[1],SevenSegLetters[5]);  //S
      DisplayADigit(dispPorts[2],SevenSegLetters[6]);  //t
      DisplayADigit(dispPorts[3],SevenSegLetters[7]);  //O
    }
  }else if(msg == "error"){
    // display best available bilingual approximation of "error"
    //  message on seven-segment display
```

```
    DisplayADigit(dispPorts[0],SevenSegLetters[1]);  //E
    DisplayADigit(dispPorts[1],SevenSegLetters[0]);  //r
    DisplayADigit(dispPorts[2],SevenSegLetters[0]);  //r
    DisplayADigit(dispPorts[3],SevenSegNumbers[12]);  //
  }
  else if(msg == "clear"){
    for(int i = 0; i < num_displays; i++){
      DisplayADigit(dispPorts[i],SevenSegNumbers[12]);
    }
  }
}

void displayForInterval(float val, String msg, long ms){
    unsigned long timer = millis();
    while(millis() - timer < ms){SevenSegDisplay(val, msg);}
    turnOffDisplay();
}

/*---------------------Interrupt and TSL230R Functions----------------------*/
void add_pulse() {pulse_count++;}
  //this simple function counts pulses sent from the sensor

void setSensitivity(int sens){    //set sensor sensitivity
  if(sens == LOW_SENSITIVITY){
    digitalWrite(TSL_S0, LOW);
    digitalWrite(TSL_S1, HIGH);
    sensitivity = LOW_SENSITIVITY;
  }else if(sens == MED_SENSITIVITY){
    digitalWrite(TSL_S0, HIGH);
    digitalWrite(TSL_S1, LOW);
    sensitivity = MED_SENSITIVITY;
  }else if(sens == HIGH_SENSITIVITY){
    digitalWrite(TSL_S0, HIGH);
    digitalWrite(TSL_S1, HIGH);
    sensitivity = HIGH_SENSITIVITY;
  }
  return;
}

float takeReadings(int num_rdgs){
  digitalWrite(IR_LED, HIGH); //turn on light source
  int rep_cnt = 0, b = 0;
  long sum = 0, low = 1000000, high = 0, rd = 0;
  displayForInterval(-1, "cycle_dashes", 1000);
  PCintPort::attachInterrupt(TSL_FREQ, add_pulse, RISING);
    //turn on frequency-counting function
  delay(200);
  pulse_count = 0; //reset frequency counter
  timer = 0;
  while(rep_cnt < num_rdgs){
    //for given number of readings
    if(millis() - timer >= SAMPLING_WINDOW){
      //once 1000 ms have elapsed
      //normalize frequency by TSL_S2 & TSL_S3 settings
      rd = pulse_count * scale_divider;
      //find highest and lowest readings in the group
      if(rd > high){high = rd;}
      if(rd < low){low = rd;}
      sum += rd;          //sum the readings
      timer = millis();     //update timer
      rep_cnt++;
      pulse_count = 0;
    }
  }
  PCintPort::detachInterrupt(TSL_FREQ);          //turn off frequency-counting function
  digitalWrite(IR_LED, LOW);                      //turn off light source
  if(num_rdgs > 3){          //chuck out highest and lowest readings and average the rest, if there are four or more readings
```

```
    sum -= (high + low);
    b = 2;
  }

  float raw_value = float(sum) / float(num_rdgs - b) / div_fact, ntu_value = -1;
    // get average reading, with highest and lowest discarded
    // for much higher turbidities, code below could easily be expanded
   // and sensitivity dynamically adjusted
  if(sensitivity == HIGH_SENSITIVITY){
    // map averaged raw sensor value to NTU
    // using calibration info stored in persistent memory
    if(raw_value > config.y4)       {ntu_value = raw_value * config.m4 + config.b4;}
    else if(raw_value > config.y3)  {ntu_value = raw_value * config.m3 + config.b3;}
    else if(raw_value > config.y2)  {ntu_value = raw_value * config.m2 + config.b2;}
    else if(raw_value > config.y1)  {ntu_value = raw_value * config.m1 + config.b1;}
    else                            {ntu_value = raw_value * config.m0 + config.b0;}
    return ntu_value;
  }else{return 9999;}
}

String baseNmap(float val){
  // baseNmap encodes turbidity values in a base64 cipher,
  // to save space if transmitting many values at once
  int value = (int)(val * 100);
  String enc = "";
  String encoding = "0123456789abcdefghijklmnopqrstuvwxyzABCDEFGHIJKLMNOPQRSTUVWXYZ/+";
  int base = encoding.length();
  encoding += encoding.substring(0,1);
  long v = value / base;
  long m = value % base;
  while (v > 0){
    enc = encoding.substring(v%base, v%base+1) + enc;
    v = v / base;
  }
  enc += encoding.substring(m%base, m%base+1);
  return enc;
}

/*------------------------------GSM Modem------------------------------------*/
//NOTE: connect the modem to pins 2 (TX), 3 (RX), 7 (RESET), 5V, and GND.
String sendMessage(char* remoteNum, String message){
  sms.beginSMS(remoteNum);  // send the message
  sms.print(message);
  sms.endSMS();
  return "complete";
}

void openConnection(){
  notConnected = true;
  while (notConnected) {
    digitalWrite(3,HIGH);       // Enable the RX pin
    if(gsmAccess.begin(PINNUMBER)==GSM_READY){notConnected = false;}
    else{delay(1000);}
  }
}

void closeConnection(){
  while(notConnected==false){
    if(gsmAccess.shutdown()==1){
      digitalWrite(3,LOW);      // Disable the RX pin
      notConnected = true;
   }
  }
}
/*--------------------------END OF PROGRAM--------------------------------*/
```

Conflicts of Interest

The authors declare no conflict of interest.

References and Notes

Please refer to the article "An Affordable Open-Source Turbidimeter" for a full list of relevant references.

© 2014 by the authors; licensee MDPI, Basel, Switzerland. This article is an open access article distributed under the terms and conditions of the Creative Commons Attribution license (http://creativecommons.org/licenses/by/3.0/).
